# Stabilization of Polymer-Polyoxometalate Coacervate Droplets by Divalent Cations

**DOI:** 10.1021/acsmacrolett.5c00839

**Published:** 2026-02-11

**Authors:** Ali Hatami, Yingxi Zhu

**Affiliations:** Department of Chemical Engineering and Materials Science, 2954 Wayne State University, 5050 Anthony Wayne Drive, Detroit, Michigan 48202, United States

## Abstract

Polymeric coacervates are two-aqueous phase separating complexes with polymer-rich dense coacervate droplets dispersed in a polymer-poor supernatant aqueous solution, which can be formed with two or more distinct polymers, including charged biomolecules and nanoclusters. Due to their ultralow interfacial tension, such coacervate droplets are inherently dynamic and unstable with a high tendency to coalescence over time. In this work, we have surprisingly found that highly concentrated divalent salts, such as CaCl_2_ and SrCl_2_, can significantly enhance the stability of dense coacervate droplets formed between a neutral polymer, poly­(ethylene glycol) (PEG) and anionic polyoxometalate (POM) nanoclusters. Dense PEG–POM coacervate droplets dispersed in CaCl_2_-added aqueous solution exhibit a robust spherical shape over a long period of time of more than two years. In comparison to the coalescent PEG–POM coacervates, the stable coacervate droplets formed in CaCl_2_ solution exhibit considerably enhanced mechanical strength with increasing POM concentration. The segregation of anionic POM nanoclusters to the outer perimetric region of the droplet with depleted PEG is observed microscopically and accounts for the strong interaction between POM and divalent cations to achieve the droplet stabilization. Such stabilized polymer-POM coacervate droplets in a simple divalent salted solution can be explored to develop a functional stable nanocolloidal dispersion with tunable compartments for applications ranging from catalysts to nanomedicines.

## Graphical abstract

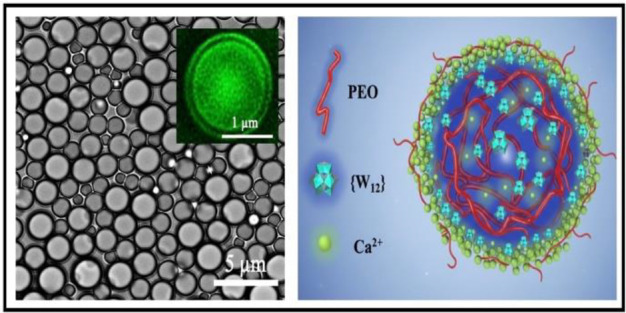

Polymer-rich complex coacervate droplets dispersed in a polymer-poor aqueous solution can be produced upon spontaneous liquid–liquid phase separation by mixing aqueous solutions consisting of two or more distinct polymers or biomacromolecules.^1–8^ The structural dynamics of dense coacervate droplets can be tuned by solution conditions, including polymer concentrations,^9^ pH,^9,10^ ionic strength,^9,11,12^ and temperature,^12,13^ leading to varied material properties of polymeric network in the dense droplets. Such dense coacervate droplets have been investigated as model membrane-free protocells to elucidate the origin of life.^14–17^ Biomimetic structured nanomaterials with responsive and coordinative behaviors have also been developed for various applications from drug delivery to battery separators and sensors.^12,18–22^ Due to the nature of two-aqueous phase separation and ultralow interfacial tension, complex coacervate droplets are inherently fluid and unstable with a high tendency to undergo Ostwald ripening and coalescence over time,^22^ which limits their applications.^23^ Different physical and chemical approaches have been explored to stabilize the coacervate droplets. For instance, chemical cross-linkers have been added to form covalent bonds with polymers inside and/or the surface of coacervate droplets.^24^ Other chemical additives, such as surfactants^25–27^ and homo- or copolymers,^28,29^ have also been explored to modify the intermolecular interactions via hydrogen bonding or hydrophobic attraction and strengthen the networking structures of the dense coacervate.^30–33^ Similar to the theme of polymersome and liposome stabilization, nanoparticles and nanowires have been applied to dress the surface of coacervate droplets for improved stability.^34^ Additionally, controlling the net charge by polyelectrolytes, pH, and ionic strength can also effectively modify the electrostatic balance and stability of coacervate droplets.^35,36^ Yet facile control and understanding of their interfacial stability of dense coacervate droplets remain considerably inadequate.

In our recent work we have investigated the complex coacervate formation between inorganic POM nanoclusters and polymers, including uncharged polymer^12,37^ and polyzwitterions^18,38^ in LiCl aqueous solution. POMs are the nanoclusters of transition metal oxides, {M_*x*_O_*y*_}_*n*_, where *n* = 4–7 and M is generally Mo, W, V, U, and Nb in well-defined crystalline structures, and carry stable and well-defined multiple negative charges over its typical nanocluster size of 1–6 nm in aqueous solution.^39–42^ We have demonstrated that the introduction of POMs can significantly enhance the viscoelasticity of gel-like POM-polymer coacervates in LiCl solution.^12,18,38^ Despite most focus of POM-polymer coacervation in monovalent salted water, it is recently reported that multivalent simple ions could lead to intriguing self-coacervation of POMs in divalent and trivalent salt solutions.^43^ In this letter, we report the study of the divalent cation effect on the coacervates formed between anionic polytungstate (Li_6_H_2_W_12_O_34_, {W_12_}) nanoclusters bearing eight negative charges over its size of 0.8 nm^44^ and uncharged PEG polymers in aqueous solutions. We have previously demonstrated that {W_12_} and PEG can form nonelectrostatic coacervates mediated by interfacial structured water as hydrogen bond donors for the PEG–water–POM association.^37^ The selection of the uncharged PEG polymer is to also reduce the complexity of multivalent counterion condensation in this study.^4,5,9,10,22,27,35^ For the interfacial water-mediated interaction, it has been historically known that such intermolecular complexation could be modified by different ions or the so-called Hoffmeister or specific ion effect.^45–48^ Thus, we experimentally examine the effect of divalent salts, CaCl_2_ and SrCl_2_, on the formation and stability of PEG–{W_12_} coacervates at varied PEG, {W_12_}, and CaCl_2_ concentrations by microscopic characterization.

We start with examining the effect of divalent CaCl_2_ salt on the phase behavior of PEG–{W_12_} coacervate formation. Following previously reported phase behavior in LiCl solution,^37^ here we focus on the modification of the phase diagram and morphological structure of PEG–{W_12_} coacervates by divalent salts. At a constant PEG35k monomer concentration, *C*_EG_ = 0.4 M, biphasic PEG–{W_12_} coacervates can be formed by mixing PEG–CaCl_2_ and {W_12_} aqueous solutions when {W_12_} concentration, *C*_{W12}_ ranges from 10 to 100 mM and CaCl_2_ concentration, *C*_CaCl2_ exceeds 0.5 M. The biphasic coacervate formation is evident by naked eyes as shown in Figure 1a and confirmed by optical and fluorescence micrographs in Figure 1b-i and ii, respectively, where fluorescence-labeled PEG, *f*-PEG is added to the PEG–CaCl_2_ solution. Upon phase separation, the dense PEG–{W_12_} coacervate droplets are dispersed in the supernatant aqueous solution similar to traditional polymer coacervation.^5,12,22,37,38,44^ At varied *C*_{W12}_ = 10–100 mM and *C*_CaCl2_ = 0.5–1.5 M and fixed *C*_EG_ = 0.4 M, the coalescence of these dense coacervate droplets is observed over elapsed time as shown in Supporting Video S1. The gradual increase of coacervate droplet size over time in Figure 1b-iii further confirms the fluid-like hallmark of biphasic coacervates.

**Figure 1:**
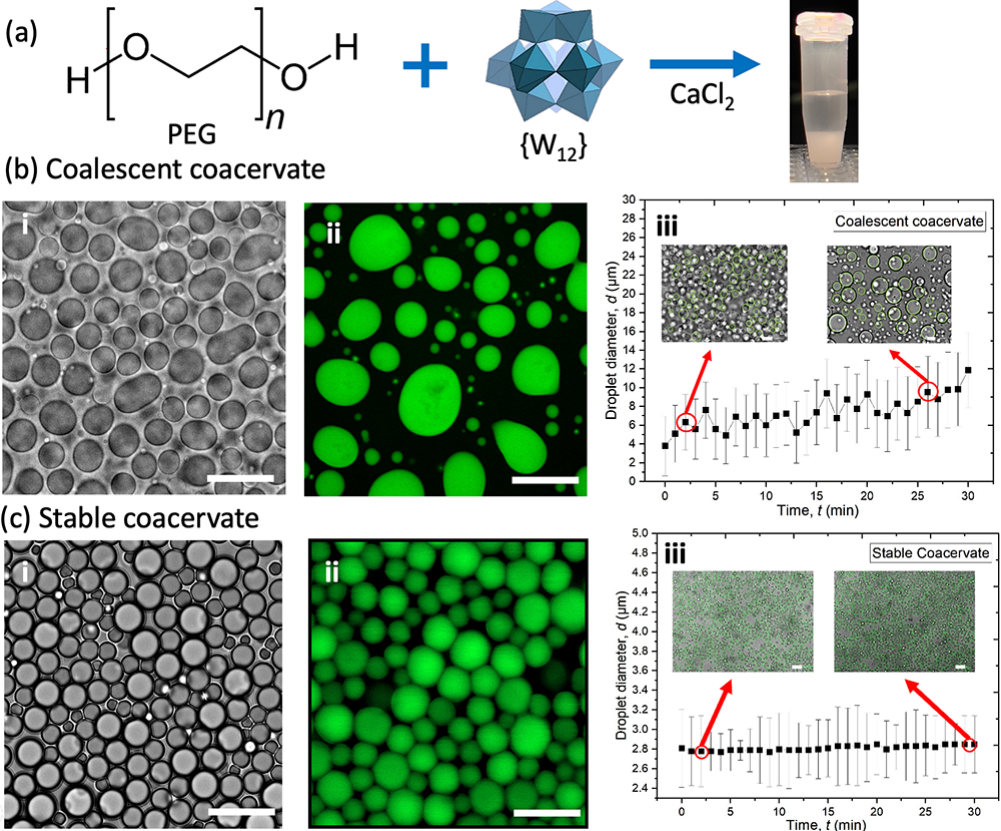
(a) Schematic illustration of the mixing of PEG and {W_12_} solutions to form biphasic PEG–{W_12_} coacervates in the vial. (b) Optical (i) and fluorescence (ii) micrographs of PEG35k-{W_12_} coacervates formed at *C*_EG_ = 0.4 M, *C*_{W12}_ = 60 mM, and *C*_CaCl2_ = 1.5 M. (iii) Average diameter of PEG–{W_12_} coacervate droplets increases over elapsed time, indicating droplet coalescence. (c) Optical (i) and (ii) fluorescence micrographs of PEG35k-{W_12_} coacervates formed at *C*_*EG*_ = 0.4 M, *C*_{W12}_ = 10 mM, and *C*_CaCl2_ = 2 M. (iii) Average droplet diameter exhibits little change over time, suggesting high stability of coacervate droplets. Insets in b-iii and c-iii: representative optical micrographs for image analysis. Scale bars in b and c are 5 μm.

However, it is most intriguing to observe that at the relatively low *C*_{W12}_ from 10 to 50 mM but at high *C*_CaCl2_ from 1.6 to 2.5 M, which approaches its upper solubility limit, the dense coacervate droplets appear to be very stable without coalescence or rupture over two years when samples are properly sealed and stored as shown in Figure 1c and Supporting Figure S1. Seemingly similar to the stability of soft colloidal particles in dense suspension,^49–51^ adjacent coacervate droplets could be in intimate contact over a short time period with flattened interfacial contact areas as evidently shown in Supporting Video S2. However, these coacervate droplets are highly stable without coalescence even during the contact period and regain their spherical shape upon being repelled apart as shown in Supporting Video S3. As shown in Figure 1c-iii, the variance of measured average size and size polydispersity of stable coacervates is <0.1% and nearly negligible over 30 min, in sharp contrast to those of traditional coalescent coacervates, which grows considerably over time as shown in Figure 1b-iii.

Despite our focus on CaCl_2_ salt due to its high water solubility^52^ and biological relevance,^53^ we have also examined other divalent salts in the same alkaline-earth group, such as SrCl_2_ and BaCl_2_. As shown in Supporting Figure S2 and Video S4, adding SrCl_2_ at *C*_SrCl2_ = 2 M to the mixture of *C*_EG_ = 0.4 M and *C*_{W12}_ = 50 mM can also lead to the formation of stable PEG–{W_12_} coacervate droplets. However, we observe no stabilization of PEG–{W_12_} coacervate droplets by adding BaCl_2_ in a similar concentration range. The full spectrum of the specific divalent ion effect^43,54,55^ on the phase diagram and stability of PEG–{W_12_} coacervation could warrant a future study.

The formation and stabilization of PEG–{W_12_} complex coacervates depend strongly on PEG, {W_12_}, and CaCl_2_ concentrations as the phase diagram at constant *C*_EG_ = 0.4 M is summarized in Figure 2a. At *C*_CaCl2_ < 0.5 M and *C*_{W12}_ < 10 mM, we observe a homogeneous clear solution upon mixing PEG–CaCl_2_ and {W_12_} solutions. At high *C*_{W12}_ > 100 mM, we observe the precipitation of PEG–{W_12_} aggregates from aqueous solution for the entirely varied *C*_CaCl2_, as shown in Supporting Figure S3. At intermediate *C*_{W12}_ = 10–100 mM and relatively low concentration *C*_CaCl2_, traditional biphasic PEG–{W_12_} coacervation with coalescent dense droplets is observed. Intriguingly, stable PEG–{W_12_} coacervate droplets are observed in the intermediate low *C*_{W12}_ = 10–50 mM but in the high *C*_CaCl2_ = 1.6–2.5 M. Considering that {W_12_} bears eight negative charges in water, we have also estimated that the stable coacervate droplets are formed in the range of ∼2–10% negative-to-positive charge ratio at *C*_CaCl2_ = 2.0 M and *C*_{W12}_ = 10–50 mM, as indicated by the boxed region in Figure 2a. Additionally, increasing *C*_EG_ can further broaden the region of stable coacervate droplets as shown in Figure 2b. With increasing *C*_EG_ from 0.4 to 1.13 M, the upper {W_12_} concentration boundary for the stable coacervate region also expands from 50 to 80 mM.

**Figure 2:**
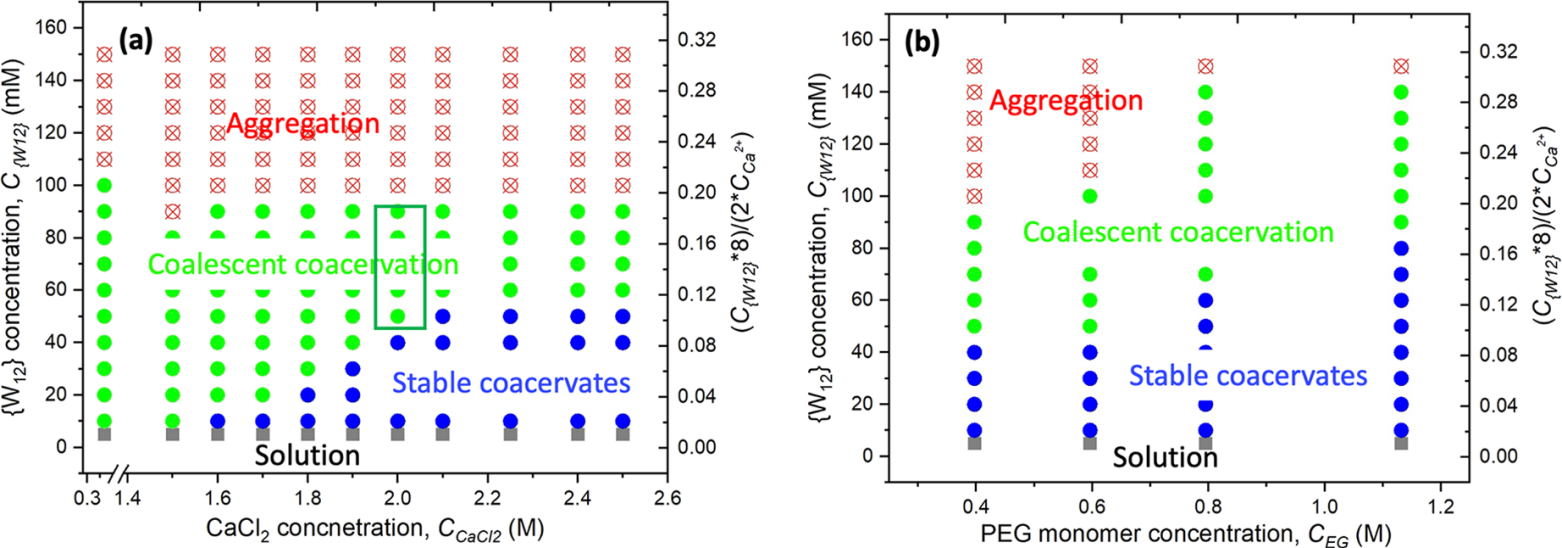
(a) Phase diagram of PEG35k–{W_12_} coacervate formation against *C*_EG_ and *C*_{W12}_ at *C*_EG_ = 0.4 M. Four distinct complex phases are observed: solid aggregates (red crossed circles), biphasic coacervates with coalescent droplets (green circles), stable coacervate droplets (blue circles), and homogeneous solution (gray squares). The charge ratio shown on the right-side *y*-axis coordinate is calculated based on constant *C*_CaCl2_ = 2.0 M for the green boxed region. (b) Phase diagram of PEG35k–{W_12_} coacervate formation against *C*_EG_ and *C*_{W12}_ at *C*_CaCl2_ = 2 M, where four distinct phase regimes are shown with the same symbols as panel (a).

Next, we have investigated the morphology of stable PEG–{W_12_} coacervate droplets deposited on a solid substrate by AFM and SEM. Surprisingly, no evident collapse or deformation of spherical coacervate droplets formed at *C*_EG_ = 0.4 M, *C*_{W12}_ = 10 mM, and *C*_CaCl2_ = 2 M is observed upon the droplet deposition on a surface during the vacuum-drying process at 85 °C overnight, as shown in Figure 3a,b. In control, the disruption of coalescent PEG–{W_12_} coacervate droplets into a random complex film is observed with the coalescent ones formed at *C*_EG_ = 0.4 M, *C*_{W12}_ = 60 mM, and *C*_CaCl2_= 1.5 M, as shown in Supporting Figure S4. The preserved spherical shape of dried PEG–{W_12_} coacervate droplets strongly suggests the high mechanical stability of these coacervate droplets formed in CaCl_2_ solutions. The size of dried coacervates is measured to be ∼0.5–1 μm by AFM and SEM, indicating 40–50% smaller than that of coacervate droplets suspended in supernatant solution due to the loss of water upon vacuum drying. More interestingly, we have observed a shell-like structure of the dried coacervate droplets in the SEM micrograph as shown in the Inset of Figure 3b. Considering no metal staining on the deposited droplets and the strong electron backscattering of {W_12_}, we attribute the outer rim with distinct and enhanced brightness to the segregation of anionic {W_12_} to the perimetric region of the droplet resulting from its strong interaction with Ca^2+^ cations in the close proximity to the droplet.^56^ Based on the measured chemical compositions as shown in Figure 3c, the presence of {W_12_} and PEG inside the droplets is confirmed by the elevated concentration profiles of tungsten (W) and carbon (C) elements in comparison to their respective concentrations outside the droplet region. In contrast, CaCl_2_ remains mostly outside the dense coacervate droplets corresponding to those of calcium (Ca) and chlorine (Cl) elements, which is consistent with the driving force of coacervation due to the release of counterions, i.e., Ca^2+^ cations, into the supernatant solution. Yet it should be noted that due to the poor spatial resolution of EDS, accurate quantification of each component concentration profile in a dense coacervate droplet is rather difficult.

**Figure 3:**
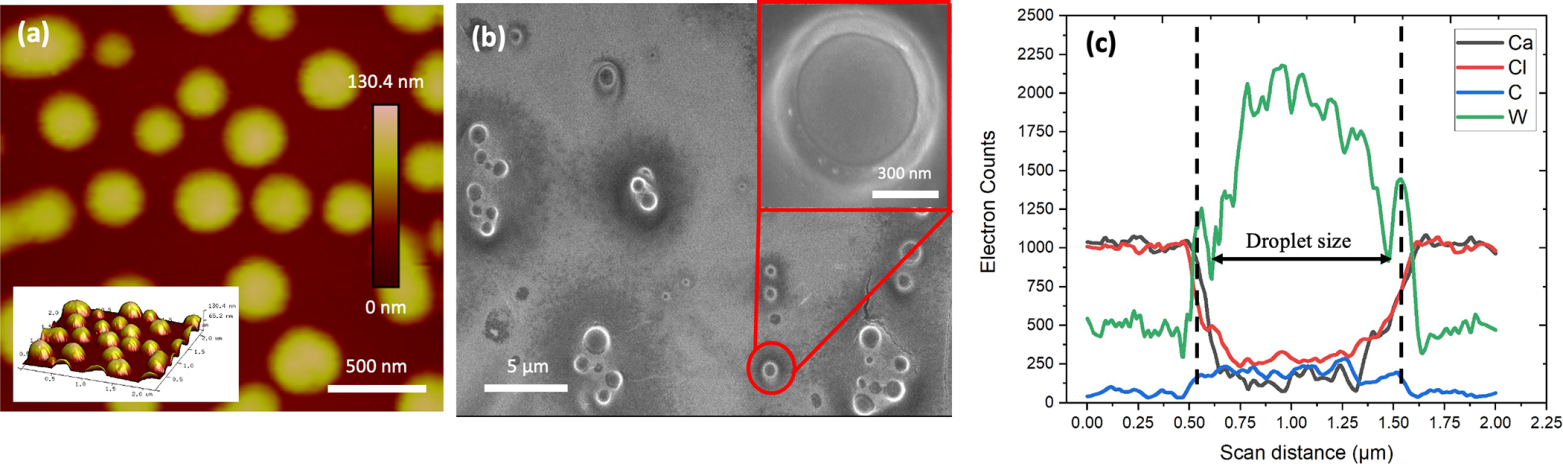
Morphology of dried dense PEG–{W_12_} coacervate droplets formed at *C*_EG_ = 0.4 M, *C*_{W12}_ = 10 mM, and *C*_CaCl2_= 2 M on a solid substrate and characterized by (a) tapping-mode AFM and (b) SEM. Inset of Panel (b): The blowout view of the SEM micrograph of a PEG–{W_12_} coacervate droplet. (c) Elemental profiles of electron counts of W (green line), C (blue line), Ca (black line), and Cl (red line) obtained by SEM-EDS line scanning at 10–20 kV.

To further examine the structure of stable PEG–{W_12_} coacervate droplets suspended in aqueous media, we have also compared the morphological structure using calcium-sensitive fluorophores added to PEG–{W_12_} coacervates in comparison to *f*-PEG labeled ones by super-resolution CLSM. It is intriguing to observe a thin dark-contrast shell in the *f*-PEG labeled coacervate droplets in Figure 4a, suggesting a PEG-depleted region near the droplet perimeter. The fluorescence-depleted region observed by CLSM appears consistent with the bright shell observed by SEM in Figure 3b, both indicating a shell thickness of *h* ∼150 nm. Furthermore, for the coacervate droplets labeled with Fluo-4 calcium indicator, strong fluorescence on the outer perimeter of the droplets is observed in sharp contrast to the nonfluorescent core as shown in Figure 4b, strongly suggesting the presence of highly concentrated Ca^2+^ at the droplet aqueous interface but not much in the center of the droplet. Such component segregation in CaCl_2_-stabilized PEG–{W_12_} coacervate droplets is also supported by the observed birefringence pattern in Figure 4c, indicating strong molecular orientation in the coacervates. In the control experiment with unstable and coalescent PEG–{W_12_} coacervate droplets, no birefringence is observed as shown in Supporting Figure S5. The birefringence pattern observed with the CaCl_2_-stabilized PEG–{W_12_} coacervates is similar to the previous report of phosphotungstate-embedded polyelectrolyte coacervates, where phosphotungstate POMs interact with polycations to form a structured interfacial layer.^57^ Combining the results in Figure 3–4, we surmise that the strong electrostatic interaction between Ca^2+^ cations and anionic {W_12_} macroions leads to the segregation of {W_12_} to the droplet interface, thereby enhancing the colloidal stability of the droplets in supernatant aqueous media. It appears that such an interfacial structure is intimately attributed to the stable coacervate droplets and disappears upon adding water to lower component concentrations toward the unstable coacervate phase as demonstrated in Supporting Video S5.

**Figure 4:**
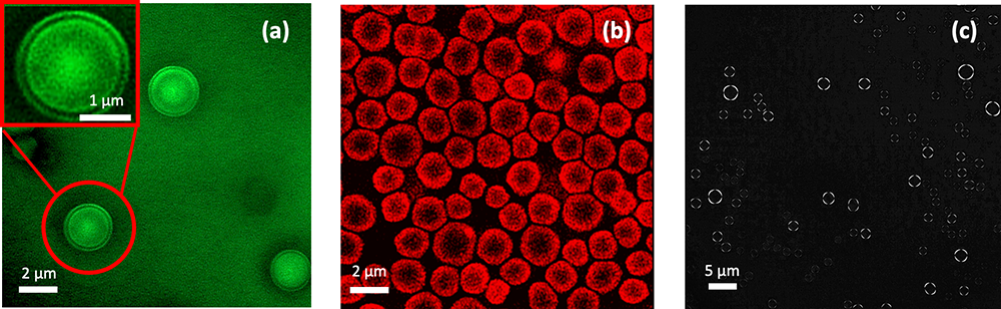
Super-resolution fluorescence micrographs of stable PEG–{W_12_} coacervate droplets in aqueous media, formed at *C*_EG_ = 0.4 M, *C*_{W12}_ = 10 mM, and *C*_CaCl2_ = 2 M and added with (a) *f*-PEG and (b) calcium-sensitive Fluo-4 fluorophores. (c) Birefringence micrograph of the stable PEG–{W_12_} coacervate droplets.

Last, but not least, we have tentatively quantified the deformation and mechanical strength of CaCl_2_-stabilized PEG–{W_12_} coacervate droplets in aqueous solution by PeakForce-mode AFM with varied loading force, *F* of an AFM probe. While their spherical shape is largely preserved as shown in Figure 5a, the height of stable PEG–{W_12_} coacervate droplets decreases with increasing *F* from 10–25 nN as shown in Figure 5b. Naively assuming no apparent adhesion between AFM probe and PEG–{W_12_} coacervate droplet, we have estimated the apparent Young’s modulus, *E*, of the droplets from the measured deformation by using the simple Hertz model^58–60^ as summarized in Figure 5c and Supporting Figure S6. The apparent *E* of PEG–{W_12_} coacervate droplets increases by nearly 1 order of magnitude as increasing *C*_{W12}_ from 10 to 30 mM, which is consistent with the effect of adding inorganic POMs on enhancing the viscoelasticity of polymer–POM complexes, including coacervates,^12,18,61^ polymer and lipid vesicles.^59,60,62,63^ However, it is noted that the *E* of stable PEG–{W_12_} coacervate droplets, ranging from 1.5–15 MPa, remains low and comparable to those of low-molecular-weight polymer vesicles^59^ and lipid vesicles in the fluid phase.^62,63^ Thus, PEG–{W_12_} coacervate droplets remain fluid and deformable, yet their colloidal stability and dispersion are significantly enhanced by the strong interaction between anionic {W_12_} macroions and Ca^2+^ cations and the resulting interfacial segregation of {W_12_}.^57,64^

**Figure 5:**
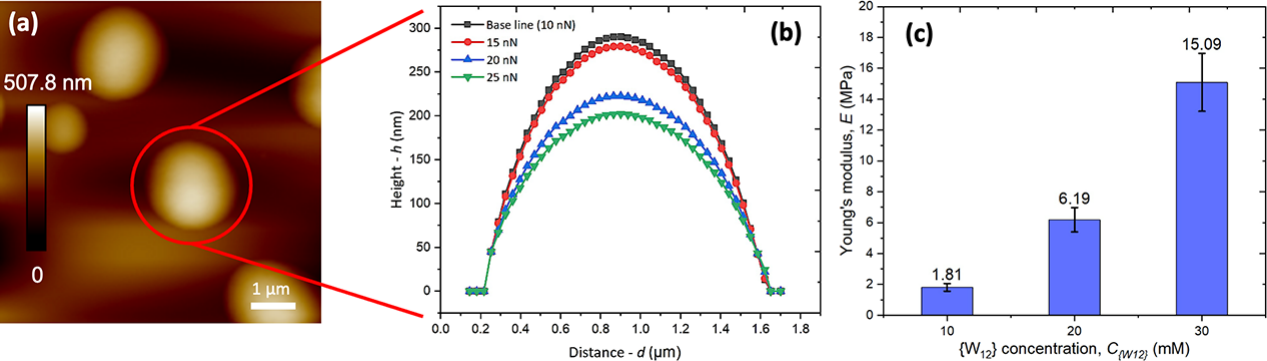
(a) Morphology of stable PEG–{W_12_} coacervate droplets deposited on a silicon wafer substrate and immersed in aqueous solution at *C*_EG_ = 0.4 M, *C*_{W12}_ = 10 mM, and *C*_CaCl2_ = 2 M under the AFM tip loading force of 10 nN. (b) Height profiles of a representative coacervate droplet under varied loading force of 10 nN (black squares), 15 nN (red circles), 20 nN (blue triangles), and 25 nN (green inverted triangles). The solid lines are the fitting results of the spherical droplet shape. (c) Apparent Young’s moduli of PEG–{W_12_} coacervate droplets formed at fixed *C*_EG_ = 0.4 M and *C*_CaCl2_ = 2 M, but varied *C*_{W12}_. Error bars are obtained with repeated measurements, with at least three independent droplets deposited on the same substrate.

In summary, we report that divalent salts can effectively enhance the stability and structure of PEG–{W_12_} dense coacervates without noticeable coalescence in aqueous solutions over a long time of more than two years, in contrast to traditional coacervates formed in monovalent salt solutions. In particular, the stable PEG–{W_12_} coacervate regime is found to be in the high *C*_CaCl2_ range from 1.6 to 2.5 M and relatively low *C*_{W12}_ range from 10 to 50 mM at a fixed PEG concentration. Increasing the PEG concentration could further broaden the stable coacervate regime. Furthermore, the segregation of {W_12_} and Ca^2+^ to the droplet aqueous interface is observed in contrast to the depletion of PEG polymers from the interface, leading to a shell-like interfacial structure. We have accounted for the strong interaction between anionic {W_12_} and Ca^2+^ for the interfacial segregation to enhance the stability of coacervate droplets. As a result, the PEG–{W_12_} dense coacervate droplets in CaCl_2_ solutions can maintain their spherical shape with moderately enhanced elasticity from 1.5 to 15 MPa with increasing {W_12_} concentration from 10 to 30 mM. Similar improved stability is also observed with PEG–{W_12_} coacervate droplets in SrCl_2_ solutions but not with other multivalent salts. Hence, the colloidal stability and elasticity of fluid-like polymer–POM coacervate droplets can be effectively controlled by specific divalent salts, which could be a reminiscent of biomolecular condensates for designing artificial cells and further expand the coacervate applications from nanoreactors to nanomedicines.

## Supplementary Material













## Notes

The Supporting Information is available free of charge on the ACS Publications Web site: https://pubs.acs.org/doi/xxx . The Supporting Information is available free of charge at https://pubs.acs.org/doi/10.1021/acsmacrolett.5c00839.

Experimental details; Supporting Figure S1, long-time stability; Figure S2, generality of stable coacervates with different divalent cations; Figure S3, PEG–{W_12_} aggregation at high *C*_{W12}_; Figures S4 and S5, control experiments of unstable coacervate droplets; Figure S6, AFM force-deformation analysis (PDF)
Video S1: Real-time microscopic video of coalescent PEG35k–{W_12_} coacervates formed at *C*_EG_ = 0.4 M, *C*_{W12}_ = 60 mM, and *C*_CaCl2_ = 1.5 M, where droplet coalescence over elapsed time is evident (MP4)
Video S2: Real-time microscopic video of stable PEG35k–{W_12_} coacervates formed at *C*_EG_ = 0.4 M, *C*_{W12}_ = 10 mM, and *C*_CaCl2_ = 2 M, confirming no coalescence of droplets even though they are concentrated into intimate contact after removal some supernatant solution (MP4)
Video S3: Real-time microscopic video in high-magnification of stable PEG35k–{W_12_} coacervates formed at *C*_EG_ = 0.4 M, *C*_{W12}_ = 10 mM, and *C*_CaCl2_ = 2 M droplet to further confirm no coalescence despite interfacial intimate contact over time (MP4)
Video S4: Real-time microscopic video of stable PEG35k–{W_12_} coacervate droplets formed in SrCl_2_-added aqueous solution at *C*_EG_ = 0.4 M, *C*_{W12}_ = 50 mM, and *C*_CaCl2_ = 2 M (MP4)
Video S5: Real-time optical microscopic video to show the transition from stable PEG35k–{W_12_} coacervate droplets to unstable ones by adding deionized water to lower component concentrations and thereby settle in the traditional coalescent coacervate phase (MP4)
