# Supplementary material for: Stabilization of Polymer-Polyoxometalate Coacervate Droplets by Divalent Cations
Source: ACS Macro Lett. 2026 Feb 11;15(3):413–9. doi: 10.1021/acsmacrolett.5c00839 (PMC13001102; doi:10.1021/acsmacrolett.5c00839)
Supplement: Supplementary file 1 [file mz5c00839_si_001.pdf]

Stabilization of Polymer-Polyoxometalate Coacervate Droplets by Divalent Cations

Ali Hatami and Yingxi Zhu*
Department of Chemical Engineering and Materials Science, Wayne State University,
5050 Anthony Wayne Drive, Detroit, Michigan 48202, USA

Supporting Information

Experimental Details

Materials and Sample Preparation. PEG polymers of molecular weight, M_w= 100,000 g/mol (PEG100k), 35,000 g/mol (PEG35k), and 10,000 g/mol (PEG10k) were obtained from Sigma-Aldrich and used directly. Amine end-functionalized methoxy PEG (PEG-NH_2) of M_w = 35,300 g/mol was purchased from Jenkem Technology USA and fluorescence labeled by Alexa Fluor 488 5-tetrafluorophenyl ester (Sigma-Aldrich) and purified by dialysis in deionized water for two weeks to obtain fluorescence-labeled PEG (*f*-PEG). An aqueous solution of $\{W_{12}\}$ at 800 mM in concentration, whose chemical structure is schematically shown in Figure 1a, was purchased from LMT Liquid and used directly. Calcium chloride ($CaCl_2$), strontium chloride ($SrCl_2$), barium chloride ($BaCl_2$), and lithium chloride (LiCl) were all purchased from Sigma-Aldrich and used directly. Fluorophores of Alexa Fluor 488 5-tetrafluorophenyl ester of excitation wavelength, λ_{ex}=488 nm and Fluo-4 calcium indicator of λ_{ex}=488 nm were purchased from Thermo Fisher Scientific and Ion Biosciences, respectively. All the aqueous solutions were prepared with deionized water (Barnstead Smart2Pure, measured resistance ≥18.2 MΩ).

For the preparation of PEG-$\{W_{12}\}$ complex coacervates in different salt solutions, we mainly focused on PEG35k while we verified the reported behavior with PEG100k and PEG10k at the same ethylene glycol (EG) monomer concentration, C_{EG}. The stock aqueous solution of $CaCl_2$ at 4 M was prepared and added to PEG aqueous solution with the resulting salt concentration ranging from 0.5 M to 3 M. It is noted that to prevent $CaCl_2$ induced aggregation and precipitation of $\{W_{12}\}$, no $CaCl_2$ is added directly to the $\{W_{12}\}$ aqueous solution; instead, $CaCl_2$ is added only to the PEG aqueous solution before mixing. Thus, the resulting $CaCl_2$ concentration, C_{CaCl2} is halved upon mixing $\{W_{12}\}$ and PEG-$CaCl_2$ aqueous solutions at an equal volume ratio and reported accordingly in this work. Other divalent salts such as $SrCl_2$ and $BaCl_2$ with varied concentrations were also examined in this work. All the aqueous solutions were prepared by using

1.0 mL and 0.2 mL pipettes (Eppendorf) with its volume uncertainty <0.005 mL. PEG−{W_{12}} complex coacervates were prepared by mixing PEG-$CaCl_2$ aqueous solution of varied initial C^i_{EG} from 0.795-2.3 M and C^i_{CaCl2} from 0.5-3 M with an equal volume of {W_{12}} aqueous solution of varied initial concentration from 10-400 mM. The mixture was vortexed vigorously for 60 seconds to ensure thorough mixing and settled for at least 15 min at room temperature before experimental characterization.

For the sample preparation for both scanning electron microscopy (SEM) and atomic force microscopy (AFM) characterization, a droplet of biphasic PEG−{W_{12}} coacervate suspension was deposited on a clean silicon wafer surface and dried in vacuum overnight without any metal staining. For AFM peak-force characterization in aqueous media, the deposited sample on a solid substrate was redispersed in an aqueous solution of the same $CaCl_2$ concentration as the biphasic coacervate formation using a clean AFM fluid cell. All the characterization was performed at least three times with the samples prepared by the same procedures to ensure the reproducibility.

Characterization The phase behavior and morphological structure of PEG−{W_{12}} complexes in different salt solutions were examined by confocal laser scanning microscopy (CLSM, Carl Zeiss, LSM 800) with 63× and 100× objective lenses (both Plan Apochromat, NA = 1.4, oil immersion) and an AiryScan detector (Carl Zeiss), in which *f*-PEG was added at an f-PEG/PEG molar ratio of 1:10 in the PEG-$CaCl_2$ solution. PEG−{W_{12}} dense coacervate droplets in comparison to the homogeneous solution mixture were also characterized by optical microscopy with 20× objective lens (Plan Apochromat, NA = 0.7, air) with a cross polarizer to examine their birefringence patterns. Additionally, the calcium-sensitive Fluo-4 indicator was added to plain PEG−$CaCl_2$ aqueous solutions to examine the presence of Ca^{2+} in the coacervates. After fluorescence labeling, the biphasic complex coacervates were centrifuged copiously to remove the supernatant aqueous solution including excess fluorophores and redispersed in the $CaCl_2$ aqueous solution. The size change of dense coacervate droplets over time was analyzed with fluorescence micrographs by image analysis using ImageJ. All the characterization reported in this work was carried out at a constant temperature, T = 22 °C.

The stability and morphological structure of PEG−{W_{12}} dense coacervate droplets were also characterized by SEM with energy-dispersive X-ray spectroscopy (EDS) (JEOL, JSM-7600F), AFM (Bruker Nano, Multimode Nanoscope IV Controller), and CLSM. AFM characterization with peak-force tapping (ScanAsyst) was conducted under both air and fluid

conditions using silicon nitride tapping-mode probes, RTESPA-300 and ScanAsyst-Fluid with respective spring constant of 40 N/m and 0.7 N/m. For the AFM characterization of the deposited coacervate droplets in $CaCl_2$ liquid media, a tapping-mode fluid cell (MTFML, Bruker Nano) with an O-ring was used after being cleaned with copious ethanol and blow-dried in a stream of nitrogen. For structural characterization, the resonance frequency of the AFM ScanAsyst fluid-mode probe in aqueous solution was fixed between 9 and 10 kHz. To determine the mechanical stiffness, $k_{droplet}$ of stable coacervate droplets, peak-force mapping AFM with a contact-mode probe was conducted with a fixed scan frequency of 2 Hz.

Long-term stability of PEG–{W_{12}} coacervate droplets.

For the samples were originally prepared at an early stage of this project as dated on June 11, 2023 and properly sealed and stored to prevent water evaporation, their morphological structures were re-examined in January 2026. We have confirmed that the PEG–{W_{12}} dense coacervate droplets remained stable and dispersed without noticeable coalescence for over two years as shown in Figure S1. Size analysis revealed an average diameter of 2.9 μm, consistent with that observed with freshly prepared coacervate samples.

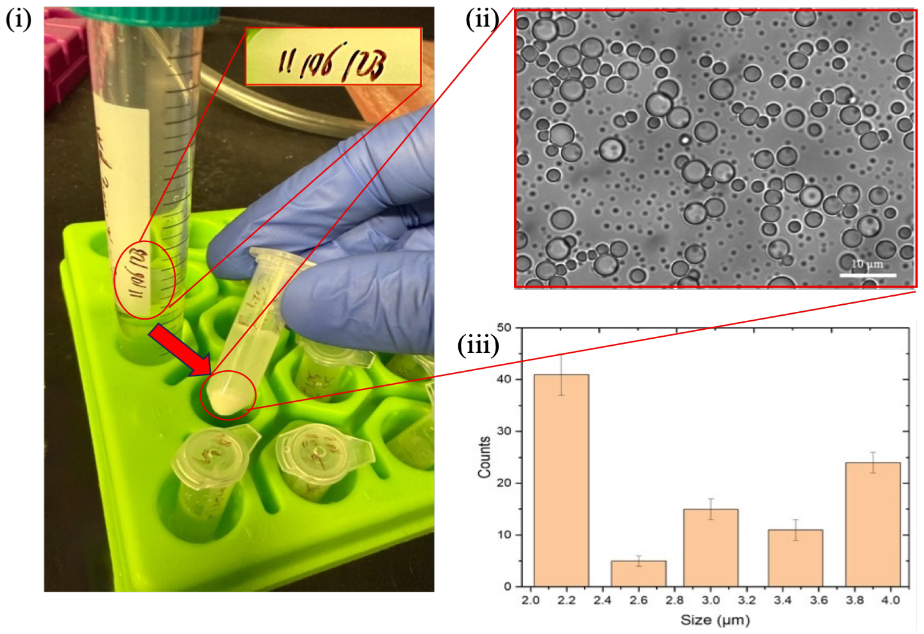

Figure S1. (i) Digital photograph and (ii) optical micrograph of stable PEG–{W_{12}} coacervates formed at C^{i}_{EG} = 0.4 mM, $C_{\{W12\}}$ = 12.5 mM, and C_{CaCl_2} = 2 M, which was prepared on June 11, 2023 and stored in a sealed vial since then and re-examined in January 2026. (iii) Size analysis shows an average droplet diameter of 2.9 μm, consistent with the average size observed with freshly prepared samples.

Generality of PEG–{W_{12}} complex coacervation with different divalent salts

To further verify the effect of divalent cations on stabilizing PEG-{W_{12}} dense coacervate droplets, we also have examined $SrCl_2$-added PEG solution in coacervation with {W_{12}} at the similar concentration conditions. Similar stable PEG-{W_{12}} coacervate droplets without coalescence over several hours are observed as shown in Figure S2 below and Supporting Video S4, confirming the generality of stabilizing PEG–{W_{12}} dense coacervate droplets by divalent cations.

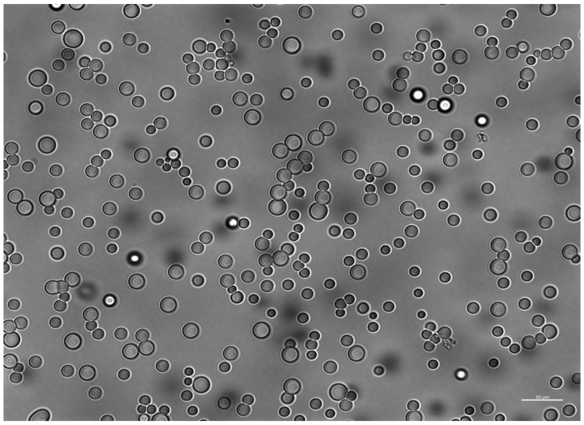

Figure S2. Optical micrograph of stable PEG35k–{W_{12}} coacervate droplets formed at C_{EG} = 0.4 M, $C_{\{W12\}}$ = 50 mM, C_{SrCl_2} = 2 M, which exhibit little coalescence over 24 hrs. Scale bar: 10 μm.

Aggregation behavior of PEG–{W_{12}} complexes at high {W12} concentrations

At relatively high {W_{12}} concentrations, $C_{\{W12\}}$ > 100 mM, the precipitation of solid PEG–{W_{12}} aggregates is observed in Figure S3, distinct from liquid-liquid phase separating coacervate regime at intermediate salt and POM concentrations.

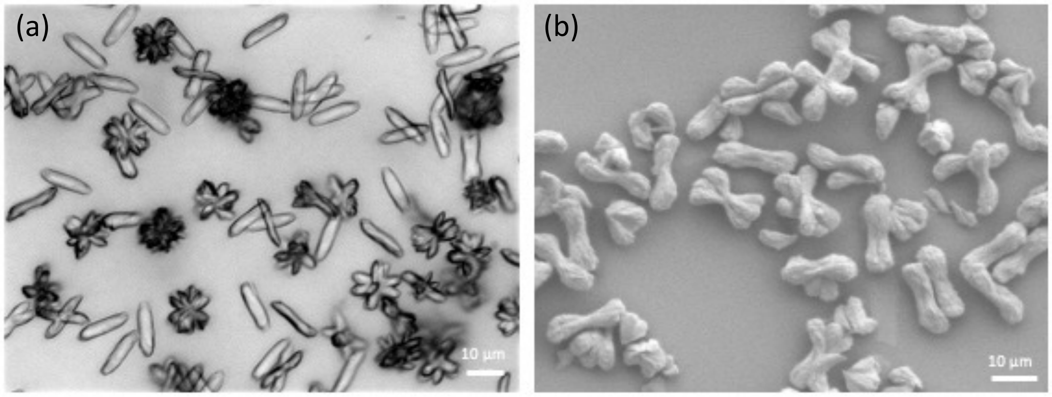

Figure S3. (a) Optical and (b) SEM micrographs show PEG35k–$\{W_{12}\}$ aggregates formed at C_{EG} = 0.4 M, $C_{\{W12\}}$ = 200 mM, C_{CaCl_2} = 2 M. SEM micrograph is obtained without any metal staining. Scale bar: 10 µm.

AFM characterization of unstable PEG–{W₁₂} coacervates

In control, we have also examined the morphology of traditional coalescent PEG–$\{W_{12}\}$ dense coacervate droplets formed at C_{EG} = 0.4 M, $C_{\{W12\}}$ = 60 mM, and C_{CaCl_2} = 1.5 M corresponding to the coalescent coacervation phase regime in Figure 2a. The droplets were deposited onto a clean silicon wafer and dried in a vacuum oven at 85 °C overnight with the same preparation as the ones shown in Figure 3-5. In contrast to the observation in Figure 3, these unstable droplets appear to collapse into random films on surface as shown in Figure S4.

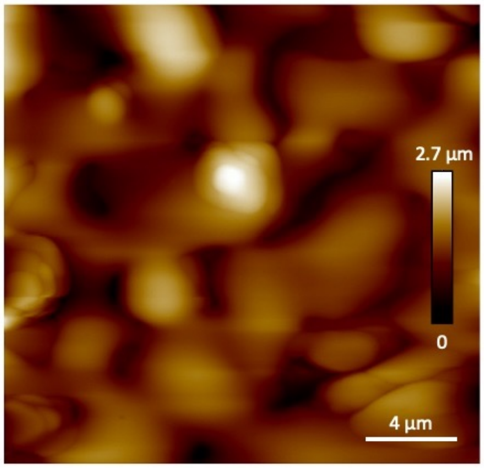

Figure S4. Micrograph of unstable PEG35k–$\{W_{12}\}$ coacervates formed at C_{EG} = 0.4 M, $C_{\{W12\}}$ = 60 mM, and C_{CaCl_2} = 1.5 M by tapping-mode AFM. Height-color scale is from 0-2.7 µm and micrograph size scale bar is 4 µm.

Birefringence comparison between unstable and stable PEG–{W₁₂} coacervates

We have also employed depolarized microscopy to examine the internal microstructural organization of PEG–$\{W_{12}\}$ dense coacervates in unstable and stable regimes. For traditional coacervates formed at C_{EG} = 0.4 M, $C_{\{W12\}}$ = 60 mM, and C_{CaCl_2} = 1.5 M, irregular shaped droplets that coalescence over time exhibit no detectable birefringence pattern, suggesting unstructured molecular microstructure inside the dense droplet as shown in Figure S5a-i-ii. In sharp contrast, for stable PEG–$\{W_{12}\}$ coacervates formed at C_{EG} = 0.4 M, $C_{\{W12\}}$ = 10 mM, and C_{CaCl_2} = 2 M, spherical droplets exhibit strong birefringence at the periphery as shown in Figure S5b-i-ii, suggesting strong molecular alignment at the interface.

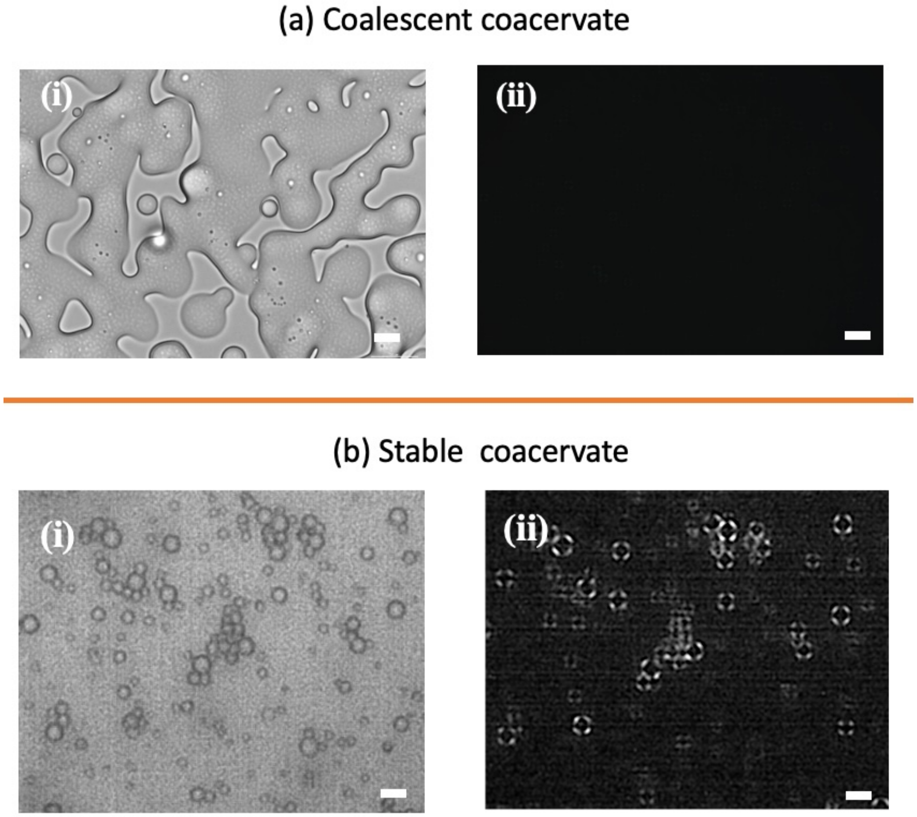

Figure S5. (i) Optical and (ii) depolarized micrograph of (a) PEG35k–{W_{12}} dense coacervates formed at C_{EG} = 0.4 M, $C_{\{W12\}}$ = 60 mM, and C_{CaCl_2} = 1.5 M, in sharp contrast to (b) the stable ones formed at C_{EG} = 0.4 M, $C_{\{W12\}}$ = 10 mM, and C_{CaCl_2} = 2 M. Scale bar in each panel is 5 µm.

Analysis of AFM force–deformation profile of PEG–{W_{12}} dense coacervate droplets

The mechanical strength of $CaCl_2$-stabilized PEG–{W_{12}} coacervate droplets in aqueous media has been characterized by PeakForce-mode AFM with a fluid cell. Specifically, the profiles of applied force, F of an AFM probe against droplet deformation, δ for stable PEG–{W_{12}} coacervate droplets formed at varied {W_{12}} concentrations are obtained. Naively assuming no apparent adhesion between AFM probe and PEG–{W_{12}} coacervate droplet, we have thereby estimated the apparent Young's modulus, E, of the droplets from the measured deformation, δ as shown in Figure 5b by using the simple Hertz model:[1-3]

$$F = \frac{4}{3} E^* R^{\frac{1}{2}} \delta^{\frac{3}{2}} \qquad \text{(Eq. 1), and}$$

$$E^* = \frac{E}{1-\nu^2} \qquad \text{(Eq. 2),}$$

where R (~20 nm) is the radius of the AFM probe, E^* is the overall Young's modulus and can be approximated to obtain E, given that the elasticity of the AFM probe is much greater than that of the coacervate droplet, and ν is the Poisson ratio (~ 0.5 in this work). With the linear fitting of applied force, F against $\delta^{\frac{3}{2}}$ as shown in Supporting Figure S6, we have obtained the E of PEG–$\{W_{12}\}$ coacervate droplets against increased $C_{\{W12\}}$ at fixed C_{EG} = 0.4 M and C_{CaCl2}= 2 M in the stable coacervate region as summarized in Figure 5c.

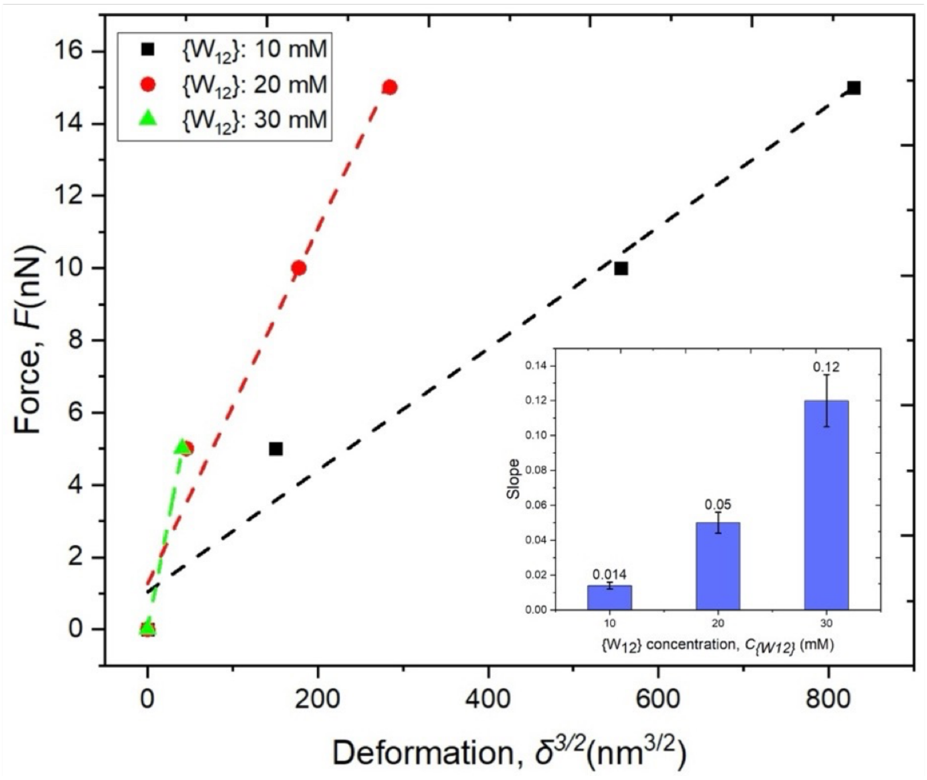

Figure S6. Measured force *(F)* vs deformation *(δ)* profiles for PEG35k–$\{W_{12}\}$ dense coacervate droplets formed at fixed C_{EG} = 0.4 M and C_{CaCl_2} = 2 M, but varied $C_{\{W12\}}$ = 10 mM (black squares), 20 mM (red circles), and 30 mM (green triangles). Inset: The slope obtained from the linear fitting, indicated by the respective dash line, of F versus $\delta^{3/2}$ is summarized against $C_{\{W12\}}$.

Supporting Video S1. Real-time microscopic video of coalescent PEG35k–$\{W_{12}\}$ coacervates formed at C_{EG} = 0.4 M, $C_{\{W12\}}$ = 60 mM, and C_{CaCl_2} = 1.5 M, where droplet coalescence over elapsed time is evident.

Supporting Video S2. Real-time microscopic video of stable PEG35k–{W_{12}} coacervates formed at C_{EG} = 0.4 M, $C_{\{W12\}}$ = 10 mM, and C_{CaCl2} = 2 M, confirming no coalescence of droplets even though they are concentrated into intimate contact after removal some supernatant solution.

Supporting Video S3. Real-time microscopic video in high-magnification of stable PEG35k–{W_{12}} coacervates formed at C_{EG} = 0.4 M, $C_{\{W12\}}$ = 10 mM, and C_{CaCl2} = 2 M droplet to further confirm no coalescence despite interfacial intimate contact over time.

Supporting Video S4. Real-time optical microscopic video of stable PEG35k–{W_{12}} coacervate droplets formed in SrCl2-added aqueous solution at C_{EG} = 0.4 M, $C_{\{W12\}}$ = 50 mM, and C_{SrCl_2} = 2 M.

Supporting Video S5. Real-time optical microscopic video to show the transition from stable PEG35k–{W_{12}} coacervate droplets to unstable ones by adding deionized water to lower component concentrations and thereby settle in the traditional coalescent coacervate phase.

References

1. Chen, Q. S., H.; Vancso, G. J. 4944−4950., Mechanical Properties of Block Copolymer Vesicle Membranes by Atomic Force Microscopy. *Soft Matter* **2009,** *5*, 4944−4950.
2. Jaskiewicz, K.; Makowski, M.; Kappl, M.; Landfester, K.; Kroeger, A., Mechanical properties of poly (dimethylsiloxane)-block-poly (2-methyloxazoline) polymersomes probed by atomic force microscopy. *Langmuir* **2012,** *28*, 12629-12636.
3. Jing, B. W., X.; Qiu, J.; Shi, Y.; Gao, H.; Zhu, Y., Shape and Mechanical Control of Poly(ethylene oxide) Based Polymersome with Polyoxometalates via Hydrogen Bond. *J. Phys. Chem. B* **2017,** *121*, 1723-1730.
